# Silencing of Transcription Factor Sp1 Promotes SN1 Transporter Regulation by Ammonia in Mouse Cortical Astrocytes

**DOI:** 10.3390/ijms20020234

**Published:** 2019-01-09

**Authors:** Katarzyna Dąbrowska, Magdalena Zielińska

**Affiliations:** Department of Neurotoxicology, Mossakowski Medical Research Centre, Polish Academy of Sciences, 5 Pawińskiego Str., 02-106 Warsaw, Poland; kdabrowska@imdik.pan.pl

**Keywords:** astrocytes, glutamine, SN1 (SNAT3), Sp1, protein kinase C, ammonia

## Abstract

The involvement of the astrocytic SN1 (SNAT3) transporter in ammonia-induced l-glutamine retention was recently documented in mouse-cultured astrocytes. Here we investigated the involvement of specificity protein 1 (Sp1) transcription factor in SN1 regulation in ammonium chloride (“ammonia”)-treated astrocytes. Sp1 expression and its cellular localization were determined using real-time qPCR, Western blot, and confocal microscopy. Sp1 binding to *Snat3* promoter was analyzed by chromatin immunoprecipitation. The role of Sp1 in SN1 expression and SN1-mediated [^3^H]glutamine uptake in ammonia-treated astrocytes was verified using siRNA and mithramycin A. The involvement of protein kinase C (PKC) isoforms in Sp1 level/phosphorylation status was verified using siRNA technology. Sp1 translocation to the nuclei and its enhanced binding to the *Snat3* promoter, along with Sp1 dependence of system N-mediated [^3^H]glutamine uptake, were observed in astrocytes upon ammonia exposure. Ammonia decreased the level of phosphorylated Sp1, and the effect was reinforced by long-term incubation with PKC modulator, phorbol 12-myristate 13-acetate, which is a treatment likely to dephosphorylate Sp1. Furthermore, silencing of the PKCδ isoform appears to enhance the ammonia effect on the Sp1 level. Collectively, the results demonstrate the regulatory role of Sp1 in regulation of SN1 expression and activity in ammonia-treated astrocytes and implicate altered Sp1 phosphorylation status in this capacity.

## 1. Introduction

l-glutamine (Gln) is the most abundant amino acid in the central nervous system (CNS) where its concentration is at least one order of magnitude higher than any other amino acid [1,2,3]. In the brain, Gln is synthesized in astrocytes from l-glutamate (Glu) and ammonia in the reaction catalyzed by glutamine synthetase [4]. The glutamate/glutamine cycle is known as a metabolite shuttle in which Gln transported from astrocytes to adjacent neurons is converted to Glu or gamma-aminobutyric acid (GABA) in an enzymatic reaction catalyzed by glutaminase or glutamate decarboxylase [4,5,6,7].

Gln is considered to play an important role in the pathogenesis of neurological diseases associated with hyperammonemia, including hepatic encephalopathy (HE), due to its excessive synthesis and accumulation in astroglial cells [8,9,10,11]. Astrocytes, the main place of ammonia detoxification in the brain, are considered as a target of ammonia-derived Gln excess [12,13]. In vitro experiments documented that in astrocytes treated with pathologically relevant concentrations of ammonia, as well as in cells exposed to a high-concentrations of Gln [14,15], create the induction of a mitochondrial permeability transition accompanied the astrocytic swelling [16,17,18]. Accordingly, astrocyte swelling is considered as a primary cause of cytotoxic component of brain edema, a frequent and fatal complication of acute HE forms [19,20]. It has also been shown that induction of mitogen-activated protein kinases (MAPK) occurs in the astroglial cells exposed to the excess of Gln [21]. In addition, intracellular Gln accumulation induces the osmotic stress and activates the p38 cascade [22,23]. Therefore, an efficient transport via specific amino acid carriers guaranteeing proper Gln inter-cellular distribution in hyperammonemic astrogliopathy is of importance.

Gln transport in astrocytes is primarily mediated by Na^+^-coupled amino acid transport system N that consists of three carriers: SN1 (aliases: SLC38A3, SNAT3), SN2 (aliases: SLC38A5, SNAT5), and SN7 (aliases: SLC38A7, SNAT7). SN1, the most abundant system N transporter, is responsible for the release of newly synthesized Gln from astrocytes [13,24,25] and its expression dominates in cultured mouse cortical astrocytes [13]. It was shown that Glu, GABA, and glycine synthesis is enhanced by the presence of SN1 [26]. The involvement and importance of SN1 in neurotransmitter recycling was confirmed by the lack of SN1 immunoreactivity in oligodendrocytes where presynaptic processes are absent [27]. Moreover, it was demonstrated that silencing of the system N transporters causes the retention of Gln in astrocytes, and that ammonia-derived Gln release from astrocytes is mediated by impairing of the SN1-mediated Gln efflux [13,25]. SN1 is abundant in the neocortex, cerebellum, and olfactory bulb; therefore, the functional ablation of this transporter with N-ethyl-N-nitrosourea (ENU)-induced mutagenesis in vivo leads to ataxia in mice [26]. This, albeit indirectly, confirms the important role of SN1 in sustaining proper neurotransmission. Therefore, investigation of the mechanisms involved in the regulation of SN1 is an important issue for understanding the Gln metabolic fates in the brain.

The *Snat3* gene promoter region possesses a well-conserved G-rich sequence with characteristics of a GT box, a potential sequence for the DNA-binding specificity protein (Sp) factors and related GT box-binding proteins [28,29]. Sp1 is a ubiquitously expressed transcription factor responsible for activation of the expression of many genes due to its ability to recruit TATA-binding protein and to fix the transcriptional start site at TATA-less promoters [30]. It is involved in many cellular processes such as cell cycle regulation, chromatin remodeling, propagation of methylation-free islands [31], apoptosis, angiogenesis, or carcinogenesis [30]. Consequently, cells lacking Sp1 are severely retarded and die after 10 days of development [31]. More recently, the literature data indicated that Sp1 interacts with SN1 in mouse liver, intestine, kidney, and HepG2 cell line. Balkrishna and colleagues [28] investigated the mechanisms underlying SN1 expression in the liver and in the brain, where SN1 levels are relatively high. It has been documented that SN1 expressing tissues contain de-methylated promoters and RNA polymerase complex occupies transcriptional start-sites, which allows activation of transcription of SN1 by Sp1 transcription factor [28]. ENCODE database indicates the enhanced activity of *Snat3* promoter in HepG2 cell line, liver, cerebellum, and brain cortex [32]. It was further shown that SN1 up-regulation occurs mainly due to enhanced binding of Sp1 to *Snat3* promoter [28]. Moreover, it was demonstrated that SN1 expression is regulated by transcription factor binding, mRNA stability and epigenetic control mechanisms. Those mechanisms mediate tissue-specific, cell-specific, and pH-specific changes of mRNA levels and further changes of protein abundance [28]. More importantly in the context of this study, an increase of Sp1 mRNA expression was previously observed in cultured rat cortical astrocytes treated for 24 h with 5 mM ammonia [33].

Sp1 is a subject of posttranslational modifications such as phosphorylation, glycosylation, and acetylation. The isoforms of protein kinase C (PKC) have been suggested to phosphorylate Sp1 as the PKC modulator phorbol 12-myristate 13-acetate (PMA) upregulate Sp1 in multiple cell types [30]. In turn, long-term exposure (12 h) of oligodendrocytes to PMA, decreases the expression of Sp1 [34]. This classical mechanism controls intracellular distribution and activity of different membrane proteins. We recently demonstrated that at the translational level SN1 transporter cells cell surface expression and activity are regulated by PKC, mainly by the PKCδ isoform in ammonia-treated astrocytes [25].

Based on the above described information and the structure of the *Snat3* gene regulatory region, we aimed to analyze the possible contribution of Sp1 on the SN1 regulation under hyperammonemic condition. We hypothesized that the mechanism by which ammonia interferes with SN1 expression may be related to the activation of Sp1 and its enhanced interaction with a *Snat3* promoter. The study also included the analysis of the role of PKC in Sp1 phosphorylation.

## 2. Results

### 2.1. Ammonia-Induced Sp1 Expression Increase and Sp1 Translocation to the Nucleus

**Figure 1:**
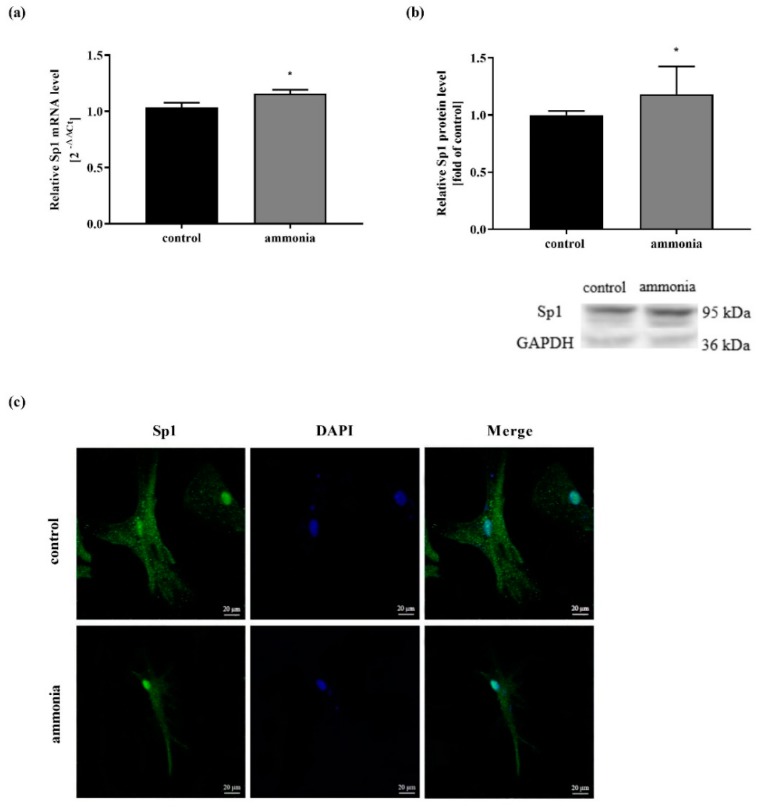
The effect of ammonia treatment on Sp1 expression and cellular localization. (**a**) Sp1 mRNA level in mouse cortical astrocytes after 24-h 5 mM ammonia treatment. Results are mean ± SD (*n* = 4). (*) *p* < 0.05 vs. control; Student t-test. (**b**) Sp1 protein level in mouse cortical astrocytes after 24-h 5 mM ammonia treatment. Upper panel shows densitometry analysis, lower panel shows representative immunoblot. Results are mean ± SD (*n* = 4). (**c**) Intracellular Sp1 transcription factor localization after 24-h 5 mM ammonia treatment (*n* = 4).

The Sp1 mRNA expression level after 24-h treatment with 5 mM ammonia was analyzed in real-time qPCR experiments. Ammonia upregulated both Sp1 mRNA and protein levels (Figure 1a,b). Since our results were in line with the results of Bodega et al. [33], the extended experiments further revealed cellular localization of the Sp1 transcription factor, as was observed using confocal microscopy. In astrocytes treated with ammonia, Sp1 was observed in cell nuclei, while in control astrocytes, the Sp1 transcription factor was observed mainly in the cytoplasm (Figure 1c).

### 2.2. Ammonia Enhances Sp1 Binding to the Snat3 Promoter Region

**Figure 2:**
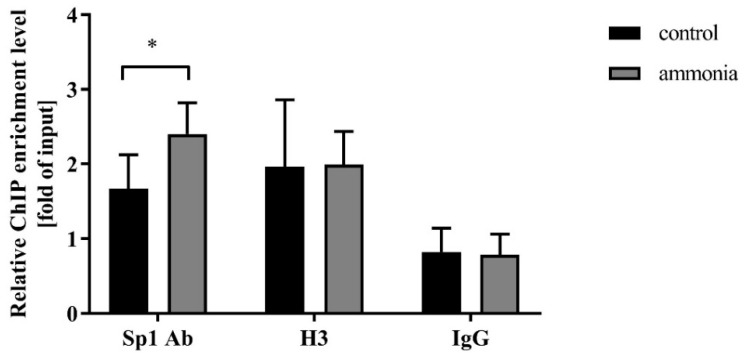
Sp1 transcription factor binding to the *Snat3* promoter region (Sp1 Ab) in mouse astrocytes treated with 5 mM ammonia for 24 h. Histone H3 was used as a positive control and IgG was used as a negative control of Sp1 binding to the *Snat3* promoter region. Results are mean ± SD (*n* = 4). (*) *p* < 0.05 vs. control; Two-way ANOVA and Bonferroni post-hoc test.

Sp1 binding activity on the *Snat3* promoter was measured using a chromatin immunoprecipitation (ChIP) assay. We observed the enrichment for the *Snat3* promoter region in both control and ammonia-treated mouse astrocytes versus the negative control IgG (Figure 2). The obtained results indicate higher enrichment for the *Snat3* promoter region in astrocytes after ammonia treatment (Figure 2).

### 2.3. Silencing of Sp1 Transcription Factor Affects SN1 Expression in the Presence of Ammonia

**Figure 3:**
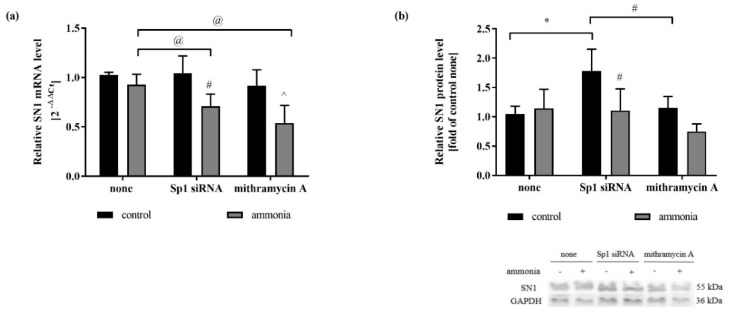
The effect of Sp1 transcription factor silencing (Sp1 siRNA) or inhibition (mithramycin A) on SN1 expression in mouse astrocytes treated or not with 5 mM ammonia for 24 h. (**a**) SN1 mRNA level. (**b**) SN1 protein level. Results are mean ± SD (*n* = 4). * *p* < 0.05 vs. control none, (@) *p* < 0.05 vs. ammonia none, (#) *p* < 0.05 vs. control Sp1 siRNA; (^) *p* < 0.05 vs. control mithramycin A; Two-Way ANOVA and Bonferroni post-hoc test.

In the next set of experiments, the expression of SN1 after silencing of Sp1 in the presence or absence of ammonia was analyzed. The knock-down of Sp1 transcription factor or its pharmacological inhibition was achieved via: (1) siRNA silencing technology (5 nM, 24 h), or (2) application of Sp1 inhibitor mithramycin A (10 µM, 1 h). The effectiveness of Sp1 silencing was checked in real-time qPCR and Western blot analyses (Figure S1). In the absence of ammonia, Sp1 silencing did not affect the SN1 mRNA expression (Figure 3a) while Sp1 silencing increased the SN1 protein level (Figure 3b). In turn, ammonia treatment specifically decreased both mRNA (Figure 3a) and protein level (Figure 3b) of SN1 in astrocytes with Sp1 transcription factor knock-down.

### 2.4. The Effect of Sp1 Transcription Factor Silencing on System N Activity in the Presence or Absence of Ammonia

Further, the verification of Sp1 involvement in the [^3^H]glutamine uptake via system N in astrocytes was conducted. Silencing of Sp1 using siRNA technology decreased the total and system N-mediated [^3^H]glutamine uptake after ammonia treatment, while Sp1 inhibition via mithramycin A affected only the total [^3^H]glutamine uptake in ammonia-treated astrocytes compared to control astrocytes after mithramycin A treatment (Table 1).

### 2.5. Sp1 Phosphorylation is Decreased in Astrocytes Treated with Ammonia

**Figure 4:**
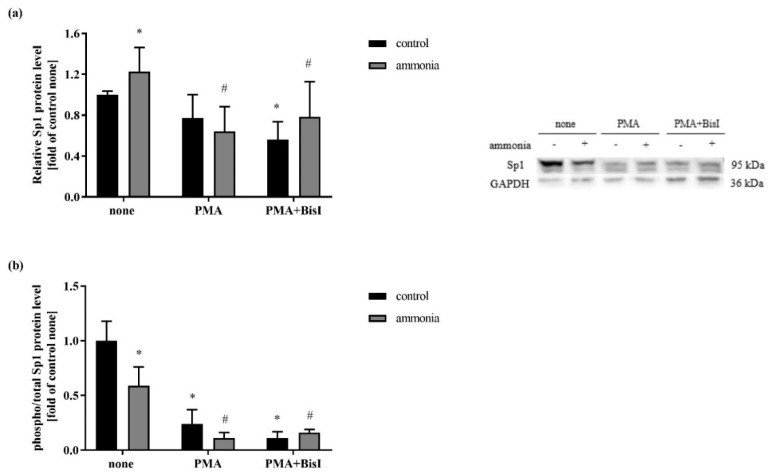
(**a**) Sp1 protein level in the astrocytes treated with 5 mM ammonia for 24 h and 200 nM PMA and/or 1 µM BisI. Results are mean ± SD (*n* = 4). (*) *p* < 0.05 vs. control none; (#) *p* < 0.05 vs. ammonia none; Two-Way ANOVA and Bonferroni post-hoc test. (**b**) The ratio of phospho-Sp1 to the total Sp1 protein level in astrocytes treated with 5 mM ammonia for 24 h and 200 nM PMA and/or 1 µM BisI. Results are mean ± SD (*n* = 4). (*) *p* < 0.05 vs. control none; (#) *p* < 0.05 vs. ammonia none; Two-Way ANOVA and Bonferroni post-hoc test.

Sp1 can be phosphorylated by different kinases such as protein kinase C (PKC) [35], protein kinase A (PKA) [36], cyclin-dependent kinase (CDK) [37], and MAPK [38]. To test the involvement of PKC in Sp1 phosphorylation, the astrocytes were exposed to 200 nM PMA, an activator of PKC [25]. To verify whether the phosphorylation of Sp1 regulates the SN1 transcription in ammonia-treated astrocytes, the Sp1 protein was analyzed via quantification of two Sp1 forms with different molecular weight. The level of Sp1 protein was determined by obtaining the ratio of the higher molecular weight, phosphorylated form with the total Sp1 protein level including both forms. As shown in Figure 4b in the astrocytes treated with ammonia, Sp1 phosphorylation was lower. When the cells undergo PMA treatment, the phosphorylation of Sp1 was further decreased. What is more, pre-treatment of astrocytes with a PKC inhibitor, bisindolylmaleimide I (BisI), did not reverse the effect induced by PMA, neither in Sp1 protein level (Figure 4a), nor in the Sp1 phosphorylation status (Figure 4b).

### 2.6. The Effect of PKC Activation on SN1 mRNA Expression in the Presence or Absence of Ammonia

**Figure 5:**
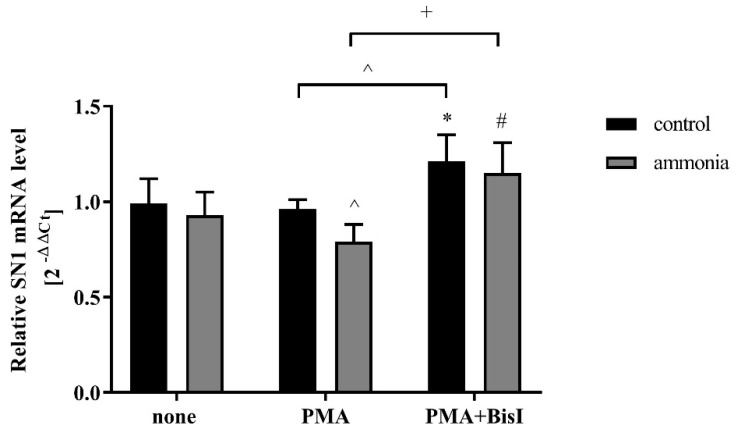
The effect of PKC activation on the SN1 mRNA level in mouse cortical astrocytes treated with 5 mM ammonia for 24 h. Results are mean ± SD (*n* = 4). (*) *p* < 0.05 vs. control none; (#) *p* < 0.05 vs. ammonia none; (^) *p* < 0.05 vs. PMA control; (+) *p* < 0.05 vs. PMA ammonia; Two-Way ANOVA and Bonferroni post-hoc test.

The incubation of astrocytes with PMA in the presence of ammonia upregulates PKCδ, but not the PKCα isoform (Figure S2). In the presence of ammonia, PMA treatment decreased the SN1 mRNA expression (Figure 5). The effect was reversed after incubation of astrocytes with a PKC inhibitor, BisI (Figure 5). Moreover, inhibition of PKC activity by BisI treatment upregulated the SN1 mRNA level (Figure 5).

### 2.7. PKCδ Isoform Depletion Abolishes Ammonia-Induced Increase of Sp1

**Figure 6:**
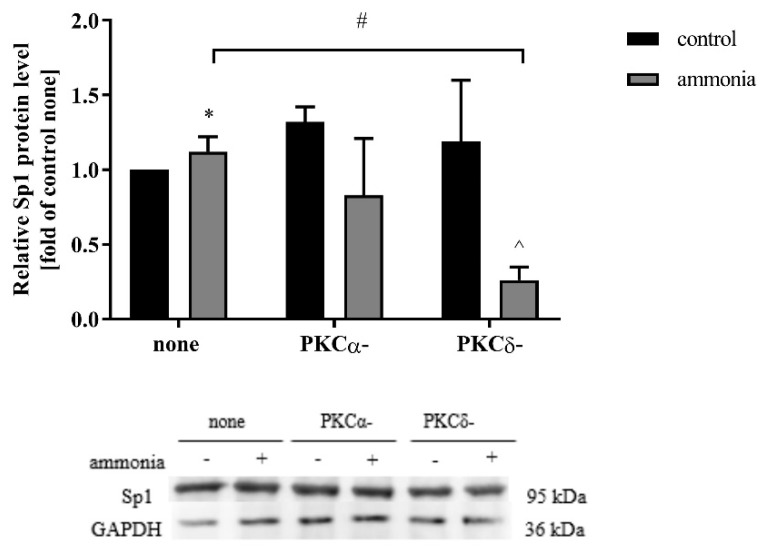
Sp1 protein level in astrocytes treated with 5 mM ammonia for 24 h and after silencing of PKCα (PKCα-) or PKCδ (PKCδ-) isoforms. Results are mean ± SD (*n* = 4). (*) *p* < 0.05 vs. control none; (#) *p* < 0.05 vs. ammonia none; (^) *p* < 0.05 vs. control PKCδ-. Two-Way ANOVA and Bonferroni post-hoc test.

According to the results described above, we aimed to identify the involvement of selected PKC isoforms in the Sp1 level. Therefore, we analyzed Sp1 protein content in ammonia-treated astrocytes with silenced PKCα and δ isoforms. Ammonia decreased the Sp1 protein level in astrocytes with silenced PKCδ isoform, while in astrocytes with silenced PKCα isoform the protein level remained unaltered (Figure 6).

## 3. Discussion

The present study demonstrates that ammonia increases the Sp1 transcription factor level and its translocation to the astrocytic nucleus (Figure 1), and implicates the above sequence of events in the regulation of SN1 transporter expression and activity in ammonia-exposed cultured cortical astrocytes (Figure 3, Table 1). The study further documents that ammonia-induced alteration in the phosphorylation status of Sp1 transcription factor may influence SN1 transporter regulation.

It has been shown that prolonged (1-, 3-, or 5-day) ammonia exposure (1, 3, and 5 mM) reduced Sp1 mRNA level in rat astroglial cells [33]. The ammonia-induced downregulation of Sp1 mRNA was inversed to the rising ammonia concentration with the maximal effect at 1 mM ammonia. Interestingly, 5 mM ammonia treatment for 1 day caused an increase of Sp1 mRNA expression [33]; however, its cellular localization has not been shown. Since Sp1 activation occurs via its nuclear translocation [33,39,40,41,42], the study was extended by the demonstration of ammonia-evoked Sp1 intracellular shift.

Pathophysiological concentrations of ammonia lead to the intracellular Gln accumulation in astrocytes, which further cause osmotic stress and activate the family members of the mitogen-activated protein (MAP) kinases [21]. Stimulation of p38MAPK triggers the increase of Sp1 expression, as has been documented [33], and its activation was further implied as a possible cause of the altered expression of different genes during HE [33,39,40,41,42].

Sp1 transcription factor is considered to be involved in the regulation of SN1 transporter in mouse kidneys during ammonia-induced acidosis [28]. The mechanism of this regulation is likely to rely on the different ability of Sp1 to bind to the DNA sequences of target genes in the presence and absence of kidney acidosis [28]. According to the available data, in the physiological condition Sp1 binds to the consensus sequences further upstream transcriptional start-site than during acidosis. Therefore, it is likely that above-described interaction causes the upregulation of SN1 transcription in acidosis, and Sp1 consensus sequence closest to the transcriptional start-site could be a stress-induced transcription factor binding site [28]. Our observations are in line with the latter finding. In our study ammonia caused the decrease of the mRNA and protein level of SN1 transporter in astrocytes with silenced Sp1 transcription factor (Figure 3a,b), suggesting that SN1 expression in astrocytes exposed to ammonia may be dependent on the Sp1 transcription factor.

The ChIP analyses of the interaction between SN1 and Sp1 transcription factor showed higher enrichment for the *Snat3* promoter region in the ammonia-treated astrocytes (Figure 2). Thus, the results implicate a stronger occupancy of this transcription factor on the promoter region of SN1 transporter in ammonia-exposed mouse astrocytes. Moreover, the interaction between SN1 and histone H3 remains unaltered upon ammonia exposure (Figure 2). This observation might be related to the decreased level of histone H3 by ammonia [43] and its role in the induction of SN1 transcription [28]. Taken together, the literature data and the observed lack of changes in the expression of SN1 after ammonia exposure may suggest that interaction of SN1 with histone H3 in ammonia-treated astrocytes remains unchanged compared to the control cells.

Although the observed changes in mRNA expression were not pronounced, it was plausible that they exert a biologically functionally significant, as it was shown previously [25,44]. Analysis of the inward [^3^H]glutamine transport in mouse astrocytes fully confirmed functional significance of the SN1 upon mechanism the Sp1 control. Indeed, ammonia decreased the total and system N-mediated [^3^H]glutamine uptake to astrocytes with silenced Sp1 transcription factor (Table 1).

It has been shown that phosphorylation of Sp1 transcription factor reversibly regulates its activity in the regenerated liver [45], where Sp1 dephosphorylation allows hepatocytes to accomplish a more proliferative cell status. In turn, Sp1 up-regulation in differentiated keratinocytes [46], caused mainly by increased level of phosphorylated form of Sp1 protein, was also induced by PKC [47]. Additionally, myelin basic protein transcription was also dependent on Sp1 phosphorylation in differentiated oligodendrocytes [34]. Considering the above evidence, we hypothesized that this mechanism may also contribute to the ammonia-induced Sp1-mediated changes in SN1. Toward the same end, our recent study showed that ammonia reduces PKC activity in cultured mouse cortical astrocytes [25]. In accordance with these findings, here we observed alterations in the Sp1 phosphorylation status in astrocytes treated with ammonia. The level of Sp1 phosphorylated form was lower in astrocytes treated with ammonia and the effect was potentiated in astrocytes exposed to PMA (Figure 4). However, co-treatment of astrocytes with the PKC inhibitor Bis I, turned out to be ineffective in reversing PMA-evoked Sp1 phosphorylation status decrease (Figure 4). The reasons for this apparent discrepancy remain to be elucidated. One other unsolved issue of our study concerns the observed decrease of Sp1 in the absence of PKCδ in ammonia-treated astrocytes. So far, the identification of the regulatory role of one selected PKC isoform has remained beyond the experimental methodology of the present study. It is worth noting that Sp1 phosphorylation could be affected not only by PKC, but also by PKA [36], DNA-dependent protein kinase [48], or CDK [37]. Clearly, the specific involvement of other kinases in ammonia-exposed astrocytes needs further evaluation. Of note in this context, Sp1 transcription factor is also regulated by other posttranslational modifications such as glycosylation and acetylation. Ubiquitous glycosylation by O-linked N-acetylglucosamine (O-GlcNAc) is considered to be analogous to phosphorylation signaling modification [49]. Sp1 glycosylation by O-GlcNAc occurs at Ser484 and translocates it to the cell nuclei leading to its activation [50,51]. However, Sp1 possess a Gln-rich trans-activating domain that contains a O-GlcNAc epitope and such modification inhibits the interactions of Sp1 with other proteins [50,52]. Moreover, acetylated on Lys703 form of Sp1 is suggested to be involved in the gene regulation by decreasing the DNA binding activity or protein interactions [53,54]. It was shown that incubation of human epidermoid carcinoma cells with PMA for more than 3 h leads to the deacetylation of Sp1 transcription factor [53]. Acetylation is related to the histone activity, which is downregulated by ammonia [43] and also by PMA treatment [53]. Since the level of histone in the cells upon such treatments is low, the acetylation of Sp1 is less likely to occur in our experimental setting than phosphorylation.

The role of the Sp1 phosphate residues in the activation of *Snat3* promoter by Sp1 is obscure. It is possible that Sp1 phosphorylation may change its interaction with other transcription factors. This phenomenon has been reported for Purα, which tended to associate with phosphorylated rather than with dephosphorylated Sp1 [55]. Further, it has been shown in cultured rat cortical astrocytes that Nrf2 transcription factor forms complexes with the transcription factor Sp1 upon the exposure to tricarbonyldichlororuthenium(II) dimer, a carbon monoxide (CO) source [56]. The complex formed, after binding to an ARE1 binding site, directly affects the regulation of Sp1 and Nrf2 target genes [56].

Our results suggest that the decrease of SN1 mRNA expression in ammonia-treated astrocytes induced by PMA (Figure 5) might be mediated, at least in part, by dephosphorylation of the transcription factor Sp1. The reason why in ammonia-treated astrocytes, the mRNA and protein level of SN1 remains unchanged despite the stronger association of Sp1 transcription factor with transporter remains to be elucidated. The obtained results are in contrast to those obtained by Balkrishna et al. (2014), which suggest Sp1 to be an enhancer of SN1 in kidney during ammonia-induced acidosis. Importantly, the transcriptional regulation of SN1 was reported as tissue-specific and predominantly controlled by various epigenetic factors [28]. In this context it is plausible that in contrast to kidney, in ammonia-treated astrocytes, Sp1 acts as a silencer of SN1. It is worth noting that recently it was demonstrated that Sp1 is a silencer of megakaryocyte-specific αIIb gene expression and mir-20b in different commercial cell lines [57,58]. Worth mentioning in this context is the postulated role of one other transcription factor, Nrf2. Recently it was shown that Nrf2 is involved in the regulation of SN1 upon metabolic acidosis in the kidney [59]. The role of Nrf2 in SN1 regulation in ammonia-treated astrocytes has not been examined as yet.

In conclusion, results of this research provide substantial evidence that pathologically elevated ammonium ions activate Sp1 transcription factor and enhance its binding to the *Snat3* promoter region, in this way contributing to the alteration in Gln uptake in cultured mouse cortical astrocytes. Moreover, the Sp1 silencing promotes SN1 regulation by ammonia. The relevance of the findings for understanding the role of Sp1 in the regulation of SN1 transporter and SN1-mediated Gln uptake in the brain during hyperammonemia in vivo remains to be documented using more native systems.

## 4. Materials and Methods

### 4.1. Materials

Plastic Corning Costar cell culture plates and cell culture medium were provided by Sigma-Aldrich (St. Louis, MO, USA), fetal bovine serum (FBS) from Biosera (Nuaillé, France), and antibiotic antimycotic from Gibco (Thermofisher Scientific, Grand Island, NY, USA). All other chemicals used in this study were of the purest grade and available from the commercial sources.

### 4.2. Astrocyte Cultures and Treatment

Primary cultures of cortical astrocytes were prepared from the 7-day-old C57BL6/J mice obtained from the animal colony of the Mossakowski Medical Research Centre, Polish Academy of Sciences in Warsaw, according to the previously described method [51]. Briefly, isolated cerebral cortex was passed through 80 µM Nitex nylon netting and then 40 µM Nitex nylon netting into Dulbecco’s Modified Eagle’s Medium (DMEM) containing 20% FBS. The medium was changed twice a week, gradually reducing to 10% FBS. To promote the morpohological differentiation of the cells, dBcAMP was added to the culture medium in the third week of culturing. The cells were grown in the atmosphere of 95% O_2_ and 5% CO_2_ at 37 °C. All experiments of this study were performed on 3-week-old cells. Astrocytes were treated with 5 mM ammonium chloride (“ammonia”, Sigma-Aldrich) [60,61] for 24 h. Moreover, in the experiments analyzing the role of PKC in the Sp1 expression, mature cells were exposed to 200 nM PMA and/or 1 µM BisI for 24 h.

### 4.3. Sp1 Transcription Factor and PKC Isoforms Silencing

The down-regulation of the Sp1 transcription factor, PKCα and PKCδ isoforms was performed via transfection of astrocytes with a mix of four types of siRNA duplexes. Each siRNA consisted of 21 nucleotides was targeted to a different gene region in order to obtain the most effective silencing. Sense strands used in this study were: (a) for Sp1 silencing: 5′-CAGCACATTTGTCACATCCAA-3′, 5′-CAGATTCTATATTATATATAT-3′, 5′-CCAGGTGATCATGGAACTCAA-3′, 5′-CAGGATGGTTC TGGTCAAATA-3′; (b) for PKCα silencing: 5′-ATGAACTGTTTCAGTCTATAA-3′, 5′-CAGGAGC AAGCACAAGTTCAA-3′, 5′-CAGCTGGTCATTGCTAACATA-3′, 5′-AAGCATTATCTTAGTG GATGA-3′; and (c) for PKCδ silencing: 5′-CCGATTCAAGGTTTATAACTA-3′, 5′-AGGGAAGACACT GGTACAGAA-3′, 5′-TTGAATGTAGTTATTGAAATA-3′, 5′-CCGGGTGGACACACCACACTA-3′. After washing with phosphate-buffered saline (PBS), three-week-old astrocytes were detached from the plates by trypsinization and then plated at a density of 1.8 × 10^5^ cells per well in six-well plates in 1.5 mL of cell culture medium (DMEM with 10% FBS). Subsequently, astrocytes were transfected with the transfection mixture consisting of 9 µL HiPerfect Transfection Reagent (Qiagen, Hilden, Germany), 4.5 µL siRNA duplexes (2 µM), and 286.5 µL OptiMEM (medium without serum; Gibco, Thermofisher Scientific, Paisley, UK) according to the fast-forward protocol designed for adherent cells provided by the manufacturer. Before drop-wise addition of the transfection mixture to the cells, it was incubated for 30 min in the room temperature to form the complexes between the reagent and siRNA. Transfected cells were cultured in the normal growth conditions for 24 h. The final concentration of siRNA in each well was 5 nM. The specificity of the silencing was checked using “mock” samples (only transfection reagent added) and negative control siRNA “AllStars” (Qiagen, Hilden, Germany) that does not silence any gene. Sp1 transcription factor was also downregulated by 1 h treatment of astrocytes with its inhibitor, mithramycin A, in the concentration of 10 µM.

### 4.4. Real-Time qPCR Analysis

One microgram of total RNA isolated from astrocytes using TRI Reagent (Sigma-Aldrich) was reverse transcribed using the High-Capacity cDNA Reverse Transcriptase Kit (Applied Biosystems, Warrington, UK). Real-time qPCR analyses were performed on 96-well plates in The Applied Biosystems 7500 Fast Real-Time PCR System using minor groove binder (MGB) Taqman probe assay and purchased from Applied Biosystems primers and probes for SN1, Sp1 and endogenous control β-actin (Mm01230670_m1, Mm00489039_m1 and Mm00607939_s1 respectively). Each reaction of total volume of 10 µL contained 5 μL TaqMan Fast Universal PCR Master Mix (Applied Biosystems), 1.5 μL of cDNA. The real-time PCR reactions were performed in the following conditions: 95 °C for 20 s followed by 45 cycles of 3 s at 95 °C and 30 s at 60 °C. The results of the analyses were calculated and expressed according to an equation (2^−ΔΔCt^) which provides the amount of the target, normalized to an endogenous reference. Ct is a threshold cycle for target amplification [52].

### 4.5. Protein Isolation and Western Blot

Astrocytes were scraped off from the plates, suspended in 1 mL of PBS, and centrifuged at 1000× *g* for 5 min at 4 °C. Then, pellets were homogenized in a RIPA buffer containing protease inhibitor cocktail (concentration 1:200, Sigma-Aldrich), phosphatase inhibitor cocktail (concentration 1:100, Sigma-Aldrich), and sodium fluoride (50 mM, Fluka, Sigma-Aldrich, Buchs, Switzerland), and centrifuged at 10,000× *g* for 10 min at 4 °C. The collected supernatant was subjected to Western blot analysis. Protein concentration was measured using a Pierce BCA Protein Assay Kit (Thermo Scientific, Thermofischer). Equal amounts of protein (30 µg) were denaturated by boiling in SDS-Page loading buffer for 10 min at 95 °C, separated on a SDS polyacrylamide gel electrophoresis and transferred onto nitrocellulose membrane. Membranes were then blocked for 1 h in 5% BSA in TBS-T buffer and incubated overnight at 4 °C in 1% BSA in TBS-T buffer with antibodies against SN1 (1:800, ProteinTech, Manchester, U.K.), Sp1 (1:500, Abcam, Cambridge, U.K.), PKCα (1:850, ProteinTech, Manchester, U.K.), PKCδ (1:850, Proteintech, Manchester, U.K.), followed by the 1-h incubation with HRP-conjugated antirabbit IgG (1:3000 for SN1, 1:5000 for Sp1 and 1:4500 for PKC isoforms; Sigma-Aldrich) for detection by Clarity Western ECL Substrate (Bio-Rad Laboratories, Hercules, CA, USA). The first antibodies were stripped off with 0.1 M glycine at pH 2.9, and membranes were incubated for 1 h at room temperature with HRP-conjugated antibody against glycerylaldehyde 3-phosphate dehydrogenase (GAPDH; 1:7500, ProteinTech, Manchester, UK). The chemiluminescent signal acquisition and densitometry analysis were performed using G-Box system (SynGene, Cambridge, UK) and GeneTools software (SynGene) respectively.

### 4.6. Gln Uptake

Cultured astrocytes were washed twice with Krebs buffer (29.5 mM NaCl, 1.13 mM KCl, 0.3 mM KH_2_PO_4_, 0.3 mM MgSO_4_, 11 mM glucose, 25 mM NaHCO_3_, 2.5 mM CaCl_2_), and pre-incubated for 15 min at 37 °C. Subsequently, the cells were incubated for 4 min at room temperature in the mixture of Krebs buffer containing 0.1 μCi/mL l-[3,4-^3^H(N)-]glutamine (PerkinElmer, Waltham, MA, USA; specific radioactivity 37 MBq/mL), 0.1 mM unlabelled Gln. In the experiments analyzing system N-mediated Gln uptake the mixture contained also 10 mM l-Ala and 10 mM l-Leu to block other Gln transporter systems [24]. The incubation was terminated by adding cold Krebs buffer with subsequent two washes using this buffer. To lyse the cells, 0.5 mL of 1 M NaOH was added and the radioactivity of cell lysates was measured in a Wallac 1409 Liquid Scintillation Counter (Perkin-Elmer, Turku, Finland).

### 4.7. Chromatin Immunoprecipitation

A total of 10^7^ cells were used for each experiment. Astrocytes were cross-linked in 1% formaldehyde for 10 min at room temperature (RT). The reaction was stopped via the addition of glycine in a final concentration of 0.125 M for 5 min. The cells were centrifuged for 3 min at 1500 rpm at 4 °C and then washed twice in cold PBS. The cell suspension was centrifuged for 5 min at 1500 rpm at 4 °C. The pellet was dissolved in a sonication buffer (10 mM Tris-HCl, pH 8; 1 mM EDTA; 0.5 mM EGTA) containing complete protease inhibitors (Roche, Mannheim, Germany), sonicated, and then centrifuged for 2 min at maximum speed at 4 °C. Supernatant was diluted in ChIP dilution buffer (0.01% SDS; 1.1% Triton-X-100; 1.2 mM EDTA; 17 mM Tris-HCl, pH 8.1; 167 mM NaCl) with an addition of protease inhibitors. The samples were incubated with salmon sperm agarose beads (Merck Millipore, Temecula, CA, USA) for 30 min at 4 °C and centrifuged (30 s, 1500 rpm, 4 °C). Fifty microliters of collected supernatant was taken as an input and the rest of supernatant was treated overnight at 4 °C either with 5 µg of Sp1 antibody (Abcam, Cambridge, U.K.), histone H3 (Cell Signaling, Leiden, The Netherlands), or control IgG (Cell Signaling, Leiden, The Netherlands). After 1-h incubation at 4 °C with salmon sperm agarose beads, the samples were centrifuged (1 min, 100 rpm, 4 °C), and the pellet was washed for 3 min in the following buffers: low salt (0.1% SDS; 1% Triton-X-100; 2 mM EDTA; 20 mM Tris-HCl, pH 8.1; 150 mM NaCl) and high salt (0.1% SDS; 1% Triton-X-100; 2 mM EDTA; 20 mM Tris-HCl, pH 8.1; 0.5 M NaCl) immune complex washing buffers, LiCl buffer (252 mM LiCl; 1% Np-40; 1% deoxycholic acid; 1 mM EDTA; 10 mM Tris-HCl, pH 8.1) and twice in TE buffer (10 mM Tris-HCl, pH 8; 1 mM EDTA). The samples were de-crosslinked by dissolving the washed beads in the elution buffer (0.1 M NaHCO_3_, 1% SDS) and addition of 5M NaCl and overnight incubation at 65 °C with shaking (950 rpm). The input samples were also prepared in the same way. The samples were incubated for 1 h at 45 °C with the mix of 0.5 M EDTA, 1 M Tris-HCl (pH 6.5), and proteinase K and then DNA from each sample was purified using a phenol:chloroform:isoamylalcohol solution (Sigma-Aldrich) with an addition of glycogen (Roche, Mannheim, Germany). The quantitative analysis of the performed experiment was checked in the real-time qPCR reactions performed using s Platinum Taq DNA Polymerase kit (Invitrogen, Carlsbad, CA, USA), 10 mM dNTPs (Invitrogen, Carlsbad, CA, USA), and SYBR Green (solution 1:2000; Invitrogen, Eugene, OR, USA). The enrichment at the *Snat3* promoter region was normalized versus input as calculated and normalized versus amylase (non-binding region). The primers used in this study were as follows: *Snat3* promoter region (sense strand: 5′-AAACACTTGGAGGGGCTTCT-3′, antisense strand: 5′-CCTCGAAATCGGTGAAGTGT-3′), amylase (sense strand: 5′-CTCCTTGTACGGGTTGTT-3′, antisense strand: 5′-AATGATGTGCACAGCTGAA-3′).

### 4.8. Immunocytochemistry

In order to investigate the cellular localization of Sp1 transcription factor in the astrocytes, the cells were cultured on poly-l-lysine coated glass coverslips in 24-well plates. The cells were washed with PBS and fixed with 4% paraformaldehyde for 20 min at RT and then permeabilized with 0.25% Triton X-100 for 15 min at RT. The cells were blocked in 3% BSA and 3% NGS (normal goat serum, Sigma-Aldrich) for 1 h. Incubation with antibody against Sp1 (1:100; ProteinTech, Manchester, UK) was done in 3% BSA and 3% NGS in PBS buffer overnight at 4 °C, and followed by 1-h incubation with goat anti-rabbit IgG Alexa Fluor 488 (1:500, Invitrogen, Waltham, MA, USA), for 1-h at RT in the dark. The cells were placed on the microscope slides using a VectaShield mounting medium containing DAPI stain (Vector Laboratories, Burlingame, CA, USA) that labelled the cell nuclei. To obtain the detailed images of the labeled cells, a confocal laser scanning microscope LSM 780 (Zeiss) was used. An argon laser (488 nm) was used for the excitation of Alexa Fluor 488 and diode 405 nm for the excitation of DAPI. Following the acquisition, the images were processed using the ZEN 2012 (Zeiss, Jena, Germany). Immunocytochemistry studies were performed in the Laboratory of Advanced Microscopy Techniques, Mossakowski Medical Research Centre, Polish Academy of Sciences.

### 4.9. Statistical Analysis

Statistical analysis was performed using GraphPad Prism 5 software (GraphPad Software, La Jolla, CA, USA). Confirmation of normality of the data distribution was checked using the Kołmogorow–Smirnow test. Statistical significance was determined using an unpaired Student’s *t*-test for two groups, one-way analysis of variance (one-way ANOVA), followed by a Dunnet post-hoc test and two-way analysis of variance (two-way ANOVA), followed by a Bonferroni post-hoc test. A probability value of 0.05 or less was considered statistically significant.

## Figures and Tables

**Table 1:** Total and system N-mediated [^3^H]glutamine uptake in mouse cortical astrocytes after 5 mM ammonia exposure for 24 h and silencing of Sp1 transcription factor by 5 nM siRNA or its inhibition by 10 µM mithramycin A. Basal [^3^H]glutamine uptake for control cells was 8.02 ± 1.43 nmol/mg of protein/min. Control—ammonia−; 5 mM ammonia treatment—ammonia+. Results are mean ± SD (*n* = 4). (*) *p* < 0.05 vs. control none (total), (^#^) *p* < 0.05 vs. control none (system N), (^&^) *p* < 0.05 vs. ammonia none (total), (^^^) *p* < 0.05 vs. control Sp1 siRNA (total), (^!^) *p* < 0.05 vs. ammonia Sp1 siRNA (total), (^+^) *p* < 0.05 vs. control Sp1 siRNA (system N), (^?^) *p* < 0.05 vs. ammonia none (system N), (^$^) *p* < 0.05 vs. control mithramycin A (system N); Two-Way ANOVA and Bonferroni post-hoc test.

Treatment	[^3^H]glutamine Uptake	Ammonia	Value (Fold of Control None)
**none**	total	−	0.98 ± 0.09
		+	1.01 ± 0.17
	system N	−	0.39 ± 0.11 *
		+	0.36 ± 0.13 ^&^
**Sp1 siRNA**	total	−	1.04 ± 0.19
		+	0.73 ± 0.12 ^^,&^
	system N	−	0.42 ± 0.06 ^^^
		+	0.29 ± 0.06 ^+,!^
**mithramycin A**	total	−	1.28 ± 0.24 *
		+	1.10 ± 0.14 ^$^
	system N	−	0.24 ± 0.10 ^#^
		+	0.15 ± 0.04 ^?^

## Supplementary Materials

It can be found at http://www.mdpi.com/1422-0067/20/2/234/s1.

## Abbreviations

BisI	bisindolylmaleimide I
CDK	cyclin-dependent kinase
ChIP	chromatin immunoprecipitation
CNS	central nervous system
dBcAMP	dibutyryl cyclic adenosine monophosphate
ENU	N-ethyl-N-nitrosourea
ERK	extracellular signal-regulated kinases
GABA	γ-aminobutyric acid
GAPDH	glyceraldehyde 3-phosphate dehydrogenase
Gln	l-glutamine
Glu	l-glutamate
HE	hepatic encephalopathy
MAPK	mitogen-activated protein kinases
O-GlcNAc	O-linked N-acetylglucosamine
PKA	protein kinase A
PKC	protein kinase C
PKCα-	silenced expression of protein kinase C isoform α
PKCδ-	silenced expression of protein kinase C isoform δ
PMA	phorbol 12-myristate 13-acetate
SN1	SNAT3, solute carrier family 38 member 3
SN2	SNAT5, solute carrier family 38 member 5
Sp1	specificity protein 1
