# Supplementary material for: Silencing of Transcription Factor Sp1 Promotes SN1 Transporter Regulation by Ammonia in Mouse Cortical Astrocytes
Source: Int J Mol Sci. 2019 Jan 9;20(2):234. doi: 10.3390/ijms20020234 (PMC6359076; doi:10.3390/ijms20020234)
Supplement: Supplementary file 1 [file ijms-20-00234-s001.pdf]

**(a)**

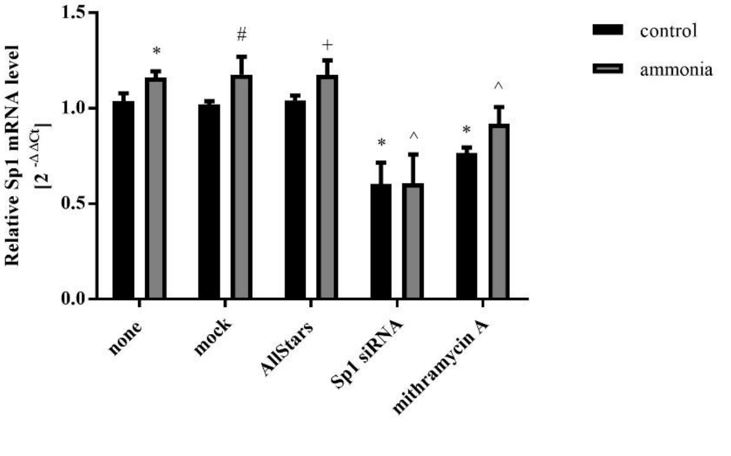


**(b)**

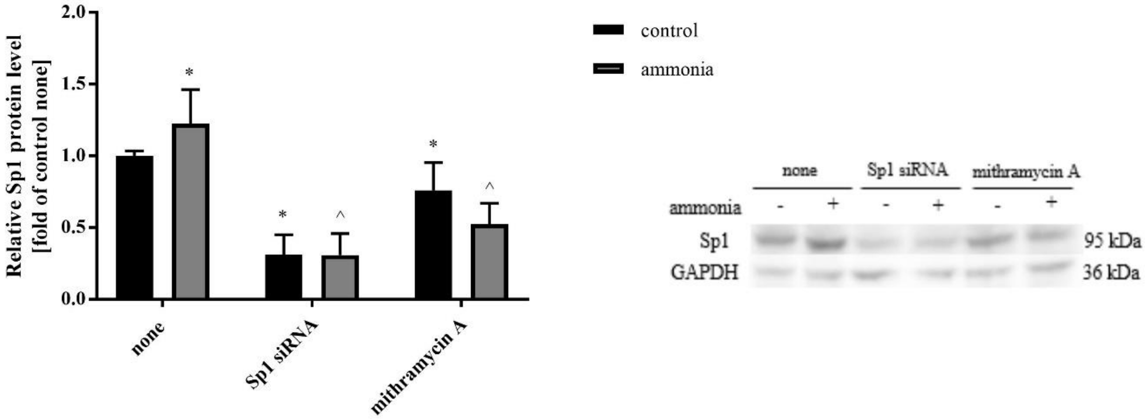


**Figure S1.** The effectiveness of Sp1 silencing by 5 nM siRNA and 10 mM mithramycin A. (**a**) Sp1 mRNA level. Mock is the transfection only with the transfection reagent; All Stars is a transfection with siRNA that does not silence any gene. Results are mean ± SD ($n$ = 4). (*) $p < 0.05$ vs control none, (^) $p < 0.05$ vs ammonia none, (#) $p < 0.05$ vs control mock, (+) $p < 0.05$ vs control AllStars negative control; Two-Way ANOVA, Bonferroni post-hoc test; (**b**) Sp1 protein level. Left panel shows densitometry, right panel shows the representative immunoblots. Results are mean ± SD ($n$ = 4). (*) $p < 0.05$ vs control none, (^) $p < 0.05$ vs ammonia none; Two-Way ANOVA, Bonferroni post-hoc test.

**a)**

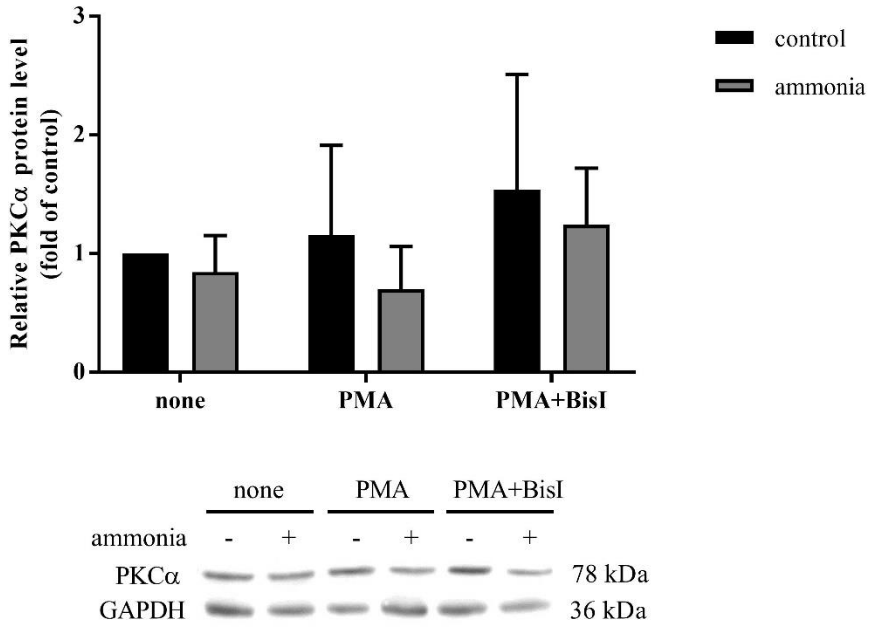


**b)**

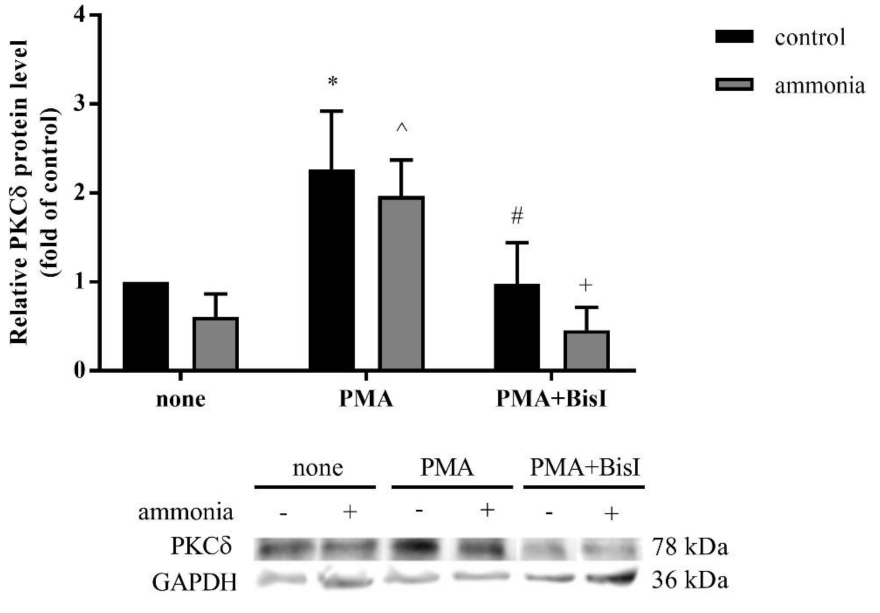


**Figure S2.** PKC isoforms *α* (**a**) and δ (**b**) protein level after 24-hour treatment with 200 nM PMA and pre-treatment with 1 mM BisI followed by 24-hour treatment with 200 nM PMA. Upper panel shows densitometry, lower panel shows the representative immunoblots Results are mean ± SD ($n$ = 4). (*) $p < 0.05$ vs control none; (^) $p < 0.05$ vs ammonia none; (#) $p < 0.05$ vs control PMA; (+) $p < 0.05$ vs ammonia PMA; Two-Way ANOVA, Bonferroni post-hoc test.
